# Atomic force microscopy: a powerful tool for high-resolution imaging of spermatozoa

**DOI:** 10.1186/1477-3155-3-9

**Published:** 2005-09-27

**Authors:** Sunil Kumar, Koel Chaudhury, Prasenjit Sen, Sujoy K Guha

**Affiliations:** 1 School of Medical Science and Technology, Indian Institute of Technology, Kharagpur 721 302, India; 2 School of Physical Sciences, Jawaharlal Nehru University, New Delhi-110067, India

## Abstract

Atomic force microscopy (AFM) has emerged as the only technique capable of real-time imaging of the surface of a living cell at nano-resolution. Since AFM provides the advantage of directly observing living biological cells in their native environment, this technique has found many applications in pharmacology, biotechnology, microbiology, structural and molecular biology, genetics and other biology-related fields. AFM has also proved to be a valuable tool for reproductive biologists. An exhaustive review on the various applications of AFM to sperm cells is presented. AFM has been extensively applied for determining the structural and topological features of spermatozoa. Unstained, unfixed spermatozoa in their natural physiological surroundings can be imaged by this technique which provides valuable information about the morphological and pathological defects in sperm cells as three-dimensional images with precise topographical details. Sperm head defects and the acrosome at the tip of the head responsible for fertilization, can be examined and correlated with the lack of functional integrity of the cell. Considerable amount of work is reported on the structural details of the highly condensed chromatin in sperm head using AFM. Detailed information on 3D topographical images of spermatozoa acquired by AFM is expected to provide a better understanding of various reproductive pathways which, in turn, can facilitate improved infertility management and/or contraceptive development.

## Introduction

Sperm morphology is regarded as a significant prognostic factor for fertilization and pregnancy [1]. Abnormal sperm morphology is one of the most common factors of male infertility. Morphological changes are also considered to be a potential target in contraceptive development. There is, therefore, an urgent need to analyze the morphological alterations of spermatozoa in their nearly physiological environment in greater detail.

Atomic force microscopy (AFM) has opened up new avenues of study in reproductive biology. AFM, invented by Binnig, Quate and Gerber in 1986, has evolved as a powerful imaging technique to obtain nanometer-resolved topographic data images. In brief, the sample surface is raster scanned by a flexible cantilever with a sharp tip at one end. A laser beam focused on the back of the cantilever is bounced off and is detected by a photodiode detector. The ability of this technique to image non-conductive living cells in physiological environment (aqueous solution) in 3D array without elaborate sample preparation or fixing of samples unlike conventional electron microscopy (which requires the cells to be fixed with aldehyde and stained) has made AFM a valuable tool to study various biomolecules [2-4], including sperm cells [5]. AFM imaging in air require cells to be fixed to avoid structural changes caused by drying forces on the cell [6]. But this fixing need not require post fixation like in electron microscopy. Conventional microscopy not only distorts sperm morphology, but is also unable to provide high-resolution 3D images owing to the small size of the spermatozoa. Optical microscopy provides valuable information only if the alterations are gross and of the order of a micron or fraction thereof.

AFM provides the advantage of directly observing spermatozoa in their native environment thereby opening the exciting possibility of analyzing their structural and functional aspects at the sub-molecular level. This article provides a review on morphological and topological images of sperm cells using AFM. Such high-resolution images are expected to provide a better understanding of male factor infertility, improve the success rate of ART procedures and also give a new direction towards contraceptive development.

## Morphological and pathological changes of spermatozoa

**Figure 1:**
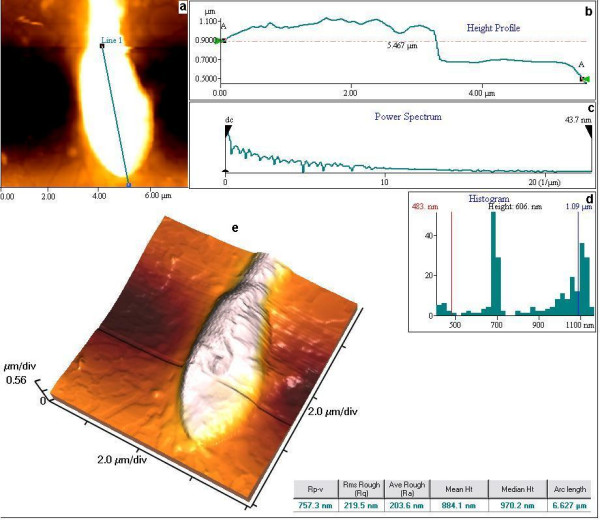
AFM of normal human spermatozoa. a. 2D image (8.00 × 8.00 μm scan) of normal sperm head. b. Height profile of the head region of spermatozoa showing clear difference in the head and the acrosomal region. c. Power spectrum of the above profile showing the scaling of roughness. d. Histogram plot of the height of the head region. e. 3D image of the head region of the spermatozoa.

Figure 1 shows the 2D and 3D images of the normal human spermatozoa using non-contact mode AFM; the graph indicates the head and the length profiles of the head region. Defects in the acrosomal region may often lead to the loss of functional competence of the spermatozoa. The major advantage of AFM in pathological studies of spermatozoa is that it allows the evaluation of position and form of the acrosome. Electron microscopy investigation reveals the presence of nano-grooves or "channels" on top of the flagellum of healthy spermatozoa [7] whereas AFM provides precise topographical information. This technique has been successfully employed for studying human sperm in its natural environment and 3D images reconstructed which enhances the contrast to resolve details such as mitochondria that surround the axoneme at the sperm middle piece [4]. An organized structure in the flagellar axoneme region in addition to depressions of the membrane that could not be observed with the conventional microscope has been reported. The 3D image contrast mechanism has been utilized to study bovine sperm cells structures [8]. Results show that imaging spermatozoa in physiologic conditions provides more native views of the cells due to the retention of cytoplasmic structures, which are otherwise easily disrupted by drying forces.

AFM has been used for morphologic and morphometric analyses of acrosome intact and acrosome-reacted human sperm heads [9]. Structural changes of the hamster sperm head surface associated with maturation, capacitation and acrosome reaction has also been studied using this technique [10]. Changes in the plasma membrane over the head region of mammalian spermatozoa during post-testicular development, after ejaculation, and after exocytosis of the acrosomal vesicle have been reported [11]. Morphological and topological alterations in human spermatozoa induced by a non-hormonal polyelectrolytic male contraceptive in vitro have been examined using AFM which suggested almost complete disintegration of the plasma membrane with subsequent rupture of the acrosomal membrane leading to dispersion of acrosomal contents [12]. A more recent study by Takano et al. (2004) provides the details of surface structure changes in spermatozoa from mouse epididymis associated with maturation [13]. Saeki et al. (2004) have provided similar details about sperm head including acrosome, equatorial segment, post acrosomal region and neck during acrosome reaction induced by lysophosphatidylcholine are given [14]. In addition, a numerical analysis carried out by the research group indicates that the area of medial sagittal plane of the anterior portions of acrosome-reacted sperm heads is approximately 40% less than those of intact heads.

Morphological alterations in spermatozoa leading to oligoasthenoteratozoospermia (OAT) and asthenozoospermia have been analyzed using AFM [15]. This study clearly indicates alteration in the infected sperms and provides extensive information on morphological changes in the head, neck and flagellum. Similar studies have shown dimensional changes in the head and defective neck and flagellum in spermatozoa from patients reporting with varicocele [16]. Recent work in this field includes the application of AFM to study the morphological and topographical changes caused by HIV and the effects of highly active antiretroviral therapy (HAART) on spermatozoon of HIV infected patients [17]. The study was so effective that even minute details, such as position of the viral particles located on the sperm membrane and their merging on the surface of spermatozoa were detected with high precision. Unlike electron microscopy, and other conventional microscopes, one of the biggest advantages of AFM is that it images virions in their nearly natural environment, which may be highly beneficial in determining interaction of virions with the host.

Detailed topology of bovine spermatozoa and force vs. distance curves has been obtained using contact mode AFM [18]. The acrosome, midpiece, postacrosomal segments and flagellum were clearly distinguishable due to the local height variations. A model of the overall mechanical response of the cell that allows separating out the mechanical response from the local surface interactions is presented. This model differs from traditional Hertzian contact models, commonly used in AFM, by explicitly taking into account the mechanics of the biomembrane and cytoskeleton [19]. With this mathematical model it is possible to determine the extent of membrane deformation due to net forces generated by the AFM tip on spermatozoa. Similar modeling is reported for analyzing deformation of living bovine spermatozoa [20]. A model to measure the mechanical response of the cells during recognition force microscopy (RFM), where specific molecules attached to the AFM tip scan the cell surface, which, in turn, provides vital information on intermolecular interaction has been proposed.

## Axonemal imaging

Axoneme, a specific "9 + 2" arrangement of the microtubules in which nine outer doublet microtubules surround a central pair of singlet microtubules, plays an important role in the movement of spermatozoa. The bending of cilia and flagella is attributed to dynein-induced sliding of microtubules, which is a key step in force generation. A transverse function of the microtubule is predicted on the basis of the 3D movement of dynein motors i.e., a motion in a direction at a right angle to the longitudinal axis of axonemes. This has been confirmed using optical trapping [21] and electron microscopy [22] which provided valuable information on the biomechanics of their movements. Recently Sakakibara et al. (2004) have successfully shown using AFM that these transverse motions occur in an oscillatory manner when the axonemes of sea-urchin sperm flagella adhere onto glass substrates [23]. They further reported that Mg-ATP significantly increases the high frequency oscillations of the flagellum. Both, a horizontal as well as a vertical component of oscillation is observed when the AFM tip is in contact with the axonemes. A similar study on the structural details and the carbon density in the flagellum of sea urchins sperm has been carried out by Tomie et al. (1991) [24].

## Sperm chromatin studies

DNA, present in a highly condensed state in mammalian sperm cells, was imaged successfully for the first time in both, air and liquids by Allen et al. (1993) [25]. The highly compact chromatin state in the sperm heads of octopus *E. cirrhosa *has been studied in great detail [26]. A simple, effective air-drying sample preparation technique for AFM of demembranated *Xenopus *sperm chromosomes has been suggested [27]. Artefact-free, high resolution nuclear reassembly images were obtained by this technique. Chromosomal banding pattern of height using AFM similar to that of G-banding by conventional optical microscopy is reported by De Grooth et al. (1992) [28]. The potential ability of AFM in localizing the DNA probes on in situ hybridized chromosomes using the height pattern has been studied. Synaptonemal complex from rat spermatocytes providing structural details of the protein has also been reported by this group.

Protamines, small arginine-rich protein, are the major DNA-binding proteins in the nucleus of spermatozoa of most vertebrates and package the DNA in a volume less than 5% of a somatic cell nucleus. The binding of protamine to sperm chromatin generates a large dense hydrophobic complex making the sperm chromatin structure difficult for microscopic examination. AFM imaging of the well-spread isolated sperm nuclei subjected to prior hypotonic treatment showed large nodular structures and a smaller nucleosome like particle near the periphery of the nucleus present in the chromatin [29]. Similarly, a toroidal shaped packaging unit for mammalian sperm chromatin has also been observed [30]. A novel method for reconstituting sperm chromatin to investigate condensation of DNA by protamine 1 is proposed [31]. Here the structures formed are found to be highly dependent on various conditions of sample preparation used for reconstitution. A previous study by Allen et al. (1992) showed that ribbon like images obtained from chromatin complexes with protamine are due to the convolutions of the imaging tip and the sample morphology [32]. A similar study on structural organisation of chromatin subunits from spermatozoa of two marsupial species, *Smithopsis crassicaudata *and *Trichosurus vulpecula *has been carried out using AFM [33]. The results indicate that the nucleohistone region consists of clusters of bigger nodules when compared to nucleoprotamine core region. A very interesting AFM study on sperm chromatin and synthetic DNA-protamine complexes is reported by Balhorn et al. (2000) [34]. The complex mimics increased resistance and structural similarity to the native sperm chromatin.

AFM has been used to perform volume measurements of the human sperm nuclei by Lee et al. (1997) [35]. Their results indicate that normal sperm and the seven of the nine classes of head-shape abnormalities studied have identical nuclear volumes, though the projected areas and shapes of the nuclei may vary widely. It is interesting to mention here that the results showed 25–40% of the sperm head morphologies found are not caused by factors that affect the volume of sperm chromatin, such as the DNA content of the sperm nucleus, differences in chromatin organization, or the extent of DNA compaction. Similar studies on volume changes in mouse and bull sperm nucleus have been carried out using scanning force microscopy by Allen et al. (1996) [36]. Their results provided details of the extent of hydration of sperm chromatin in its native state. Hence, volume of natively hydrated sperm nuclei can be easily determined.

## Future Prospects

Constant force applied on the soft biological samples may damage the cell and thus change its morphology. Considering this, various imaging modes have been developed such as resonance based tapping mode, lift mode, force modulation imaging, nanoindenting, scratching and lateral force microscopy. The development of small micro-mechanized cantilever and optical fiber tips reduce thermal noise by providing a better ratio of cantilever stiffness and resonance frequency and improved imaging bandwidth. An important development would be the construction of antibody modified tips which could be useful in localizing antigens (by vertical/lateral force detection) on the plasma membrane. This may be helpful in studying molecular interactions in greater detail.

AFM, in itself, has proved to be a powerful instrument in nanoscopic analysis of biological samples. Nevertheless, more information with finer details may be achieved if combined with various other techniques such as optical microscope [37] and optical tweezers [38] as these techniques allow direct manipulation of individual cell. Advances in Cryo-AFM are very promising for imaging spermatozoa preserved in liquid nitrogen [39,40]. This technique, in combination with freeze-etching and freeze-fracture techniques, may be used to obtain high resolution images of preserved spermatozoa.

Time lapse AFM imaging has been used to observe the conformational changes in supercoiled DNA [41] and in chaperone complex (GroEl-GroEs) analysis [42]. Small cantilevers with high resonance frequencies have been developed by Walters et al. (1996) [43]. In addition, small spring constants and electronic devices of wide bandwidth have been included in AFM to obtain a powerful useful movie mode for scanning biomolecules successively in aqueous solution [44]. This sophisticated imaging mode may be applied for a better understanding of sperm oocyte interaction.

Scanning Near-field Optical Microscope (SNOM) is an emerging technique and is still in infancy with respect to the imaging of biological cells. This technique utilizes the near field, non-propagating component of light to scan the samples with optical tips, which can be applied in the tapping or contact mode. A resolution of few tens of nanometers is achievable by SNOM. High resolution topographic and optical images of sea urchin sperm flagellum have been obtained using fluorescent probe as a light [45]. Electrostatic Force Microscopy (EFM), Magnetic Force Measurements (MFM) and Scanning Thermal Microscopy (SThM) are relatively new techniques and may play a significant role in determining fluid dynamics and biomechanics of the sperm cells in their natural micro-environment. AFM, in combination with surface potential spectroscopy, has been applied to measure the surface charges of *P. falciparum *merozoites [46]. This methodology can also be applied to study the negative charge distribution on the sperm head, which is known to play a vital role in fertilization.

Recent developments in AFM have made it a powerful tool for analyzing biomolecules. Over the last decade, AFM has emerged as a valuable technique with extensive applications in the field of sperm biology. AFM is expected to provide a better understanding of various biological pathways and intermolecular interactions which will open up new avenues in reproductive medicine.

## Authors' contributions

SK has contributed to the acquisition of a part of the data by carrying out AFM experimental studies on spermatozoa treated with the contraceptive, RISUG. PS has also assisted in acquiring data and analyzing it. In addition, he has done extensive literature survey and collected the data/research papers. KC has conceived the study and contributed to the design, analysis, co-ordination and interpretation of data. SKG, the inventor of RISUG, has also participated in the design, revised the manuscript critically for important intellectual content and has given final approval of the version to be published. All authors read and approved the final manuscript.
